# The potential of native and engineered Clostridia for biomass biorefining

**DOI:** 10.3389/fbioe.2024.1423935

**Published:** 2024-08-16

**Authors:** Paola Ponsetto, Emilia Malgorzata Sasal, Roberto Mazzoli, Francesca Valetti, Gianfranco Gilardi

**Affiliations:** Structural and Functional Biochemistry, Laboratory of Proteomics and Metabolic Engineering of Prokaryotes, Department of Life Sciences and Systems Biology, University of Torino, Torino, Italy

**Keywords:** hydrogen, ethanol, lactate, propanediol, butanol, isobutanol, medium chain esters, hexanol

## Abstract

Since their first industrial application in the acetone-butanol-ethanol (ABE) fermentation in the early 1900s, Clostridia have found large application in biomass biorefining. Overall, their fermentation products include organic acids (e.g., acetate, butyrate, lactate), short chain alcohols (e.g., ethanol, n-butanol, isobutanol), diols (e.g., 1,2-propanediol, 1,3-propanediol) and H_2_ which have several applications such as fuels, building block chemicals, solvents, food and cosmetic additives. Advantageously, several clostridial strains are able to use cheap feedstocks such as lignocellulosic biomass, food waste, glycerol or C1-gases (CO_2_, CO) which confer them additional potential as key players for the development of processes less dependent from fossil fuels and with reduced greenhouse gas emissions. The present review aims to provide a survey of research progress aimed at developing *Clostridium*-mediated biomass fermentation processes, especially as regards strain improvement by metabolic engineering.

## 1 Introduction

**FIGURE 1:**
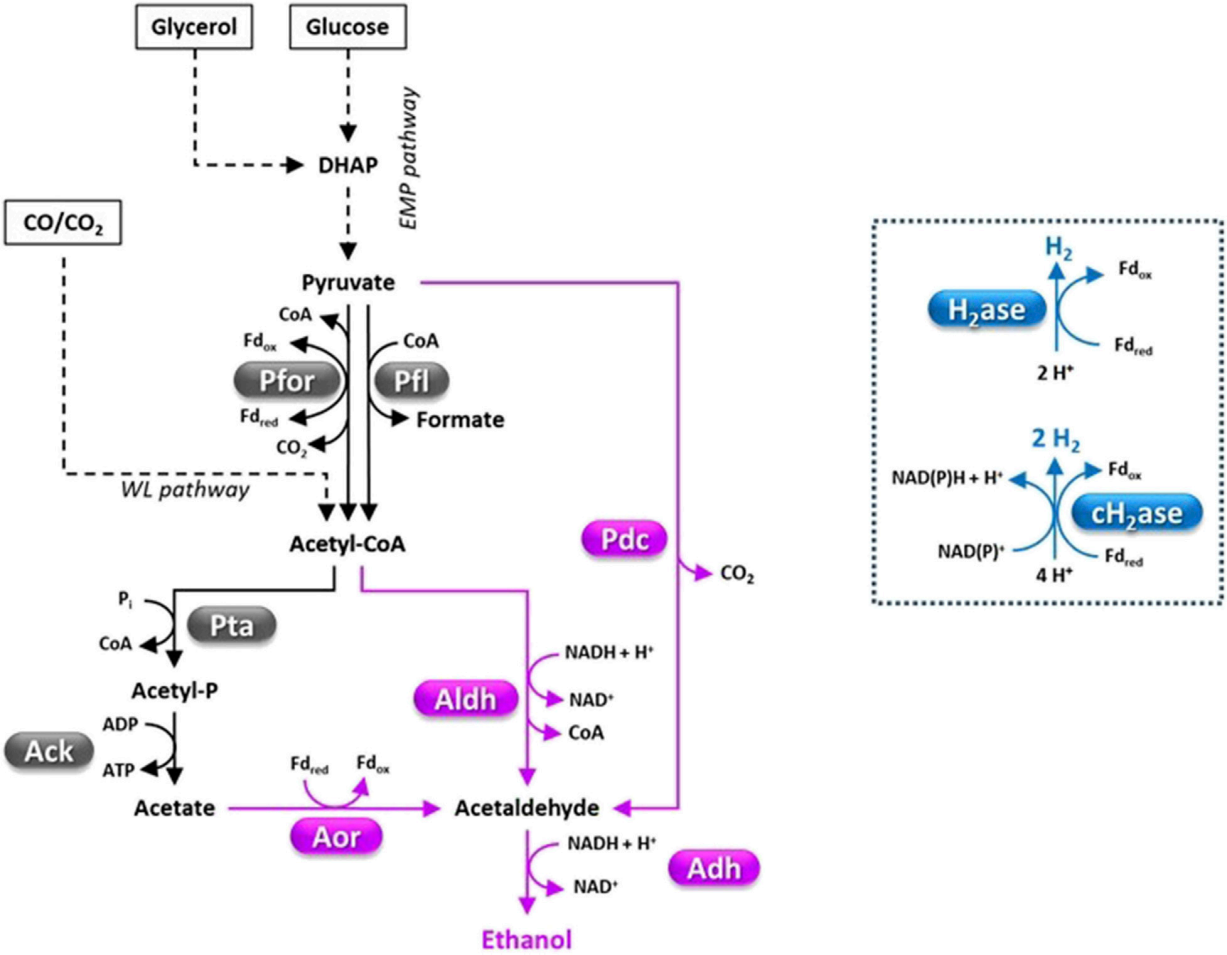
Fermentative pathways in Clostridia, production of C2-compounds and H_2_. Pyruvate decarboxylase (Pdc) has mainly been engineered in Clostridia (Tian et al., 2017a). However, a *pdc* gene has been identified on the pSOL1 megaplasmid of *C. acetobutylicum* (Lehmann and Lütke-Eversloh, 2011). Abbreviations: Acetyl-P, acetyl phosphate; Ack, acetate kinase; Adh, alcohol dehydrogenase; Aldh, aldehyde dehydrogenase; Aor, acetaldehyde ferredoxin oxidoreductase; cH_2_ase, confurcating hydrogenase; DHAP, dihydroxyacetone phosphate; EMP pathway, Embden Meyerhof Parnas pathway; Fd, ferredoxin; H_2_ase, hydrogenase; Pdc, pyruvate decarboxylase; Pfl, pyruvate formate lyase; Pfor, pyruvate ferredoxin oxidoreductase; Pta, phosphotransacetylase; WL pathway, Wood Ljungdahl pathway.

**FIGURE 2:**
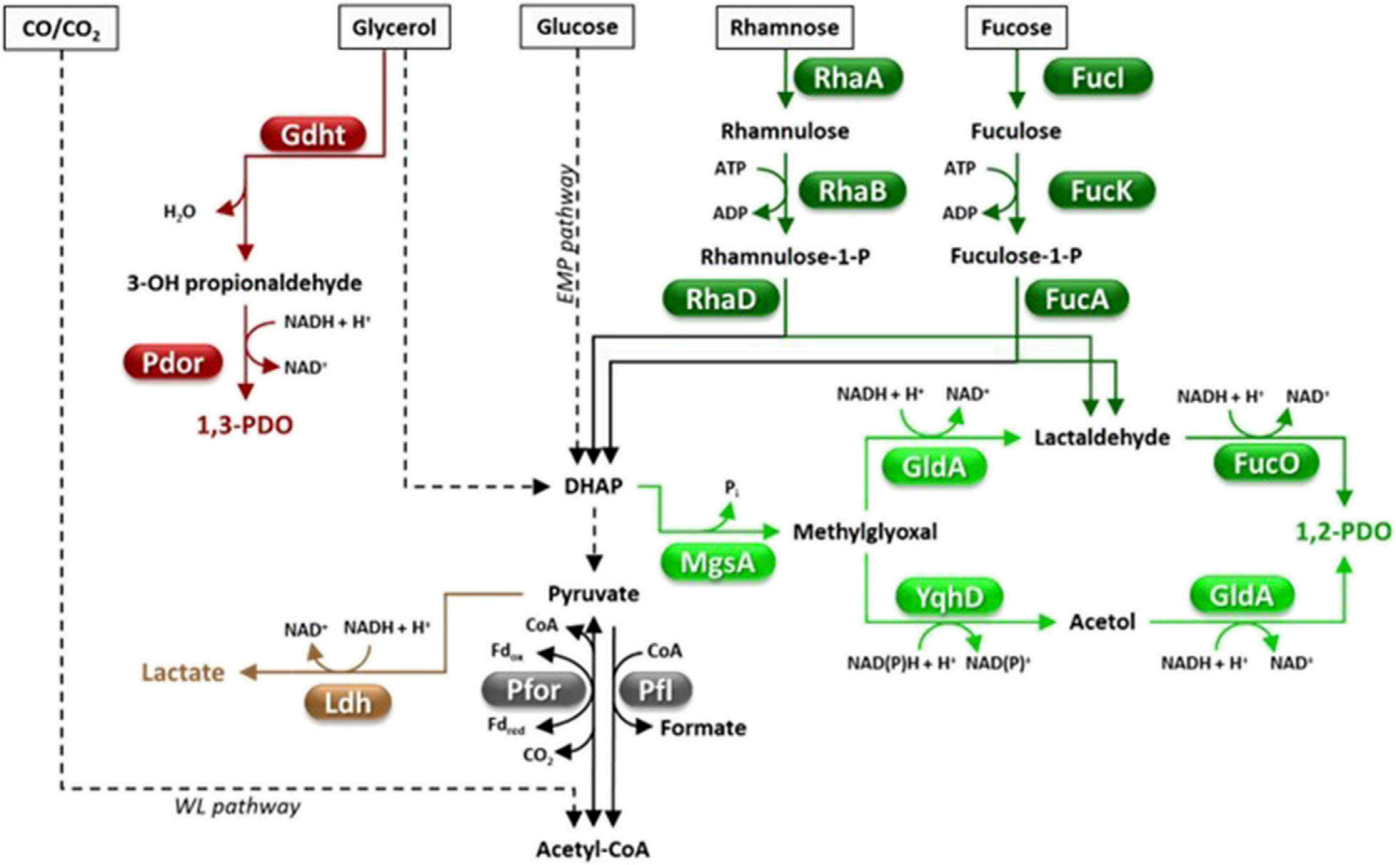
Fermentative pathways in Clostridia, production of C3-compounds. 1,2-PDO biosynthesis can occur through the methylglyoxal (light green) or the deoxyhexose pathway (dark green). Abbreviations: 1,2-PDO, 1,2-propanediol; 1,3-PDO, 1,3-propanediol; DHAP, dihydroxyacetone phosphate; EMP pathway, Embden Meyerhof Parnas pathway; Fd, ferredoxin; FucA, fuculose-1-phosphate aldolase; FucI, fucose isomerase; FucK, fuculokinase; FucO, 1,2-PDO oxidoreductase; Gdht, glycerol dehydratase; GldA, glycerol dehydrogenase; Ldh, lactate dehydrogenase; MgsA, methylglyoxal synthase; Pdor, 1,3-propanediol oxidoreductase; Pfl, pyruvate-formate lyase; Pfor, pyruvate ferredoxin oxidoreductase; RhaA, rhamnose isomerase; RhaB, rhamnulokinase; RhaD, rhamnulose-1-phosphate aldolase; WL pathway, Wood Ljungdahl pathway; YqhD, aldehyde reductase.

**FIGURE 3:**
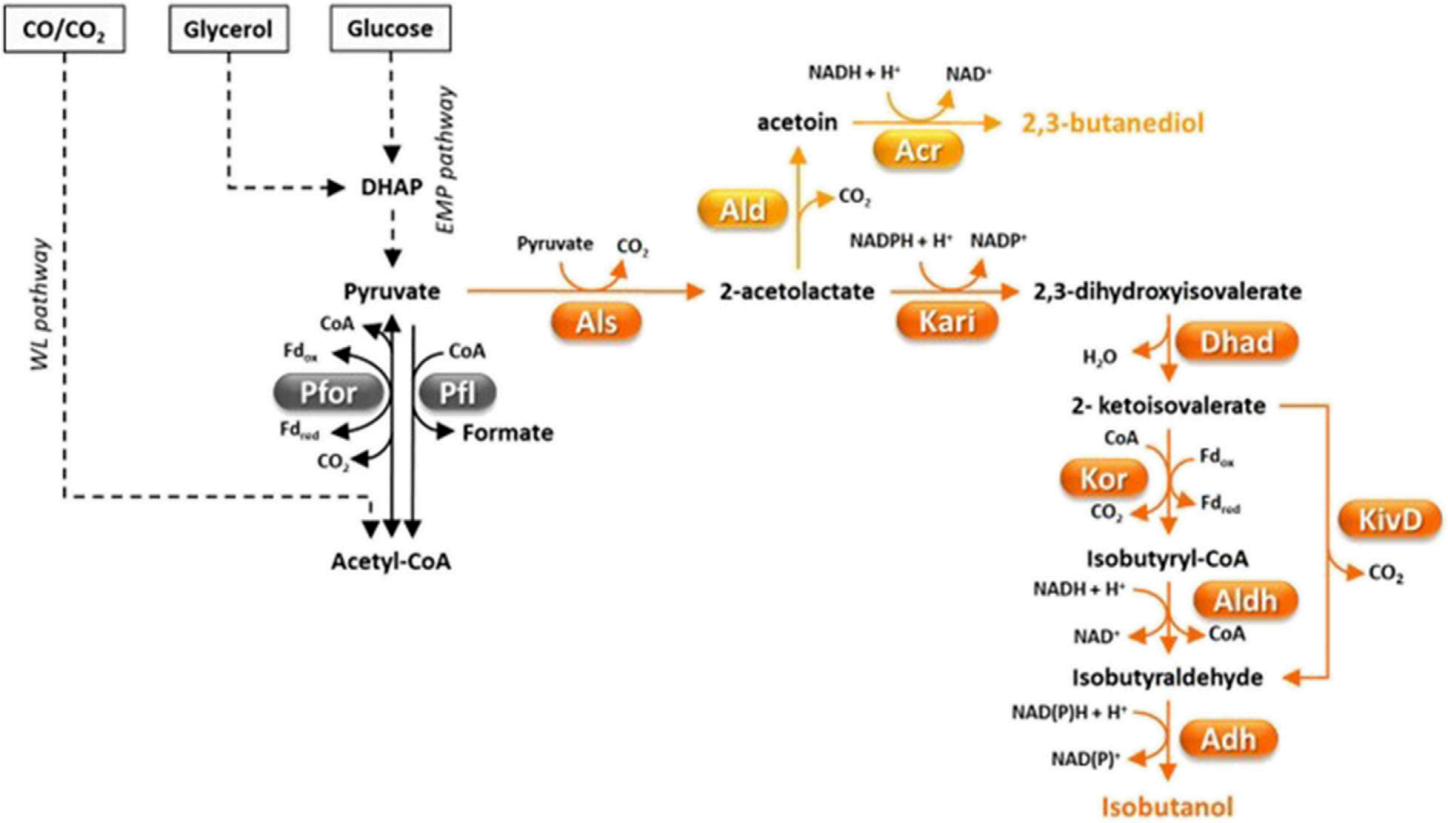
Fermentative pathways in Clostridia, production of C4-compounds. 2-ketoisovalerate decarboxylase (KivD) was not found in Clostridia but a *Lactococcus lactis* gene coding for it was engineered in *C. thermocellum* (Lin et al., 2015). Abbreviations: Acr, acetoin reductase; Adh, alcohol dehydrogenase; Ald, α-acetolactate decarboxylase; Aldh, aldehyde dehydrogenase; Als, α-acetolactate synthase; Dhad, dihydroxy acid dehydratase; DHAP, dyhydroxyacetone phosphate; Fd, ferredoxin; Kari, keto acid reductoisomerase; KivD, *L. lactis* 2-ketoisovalerate decarboxylase; Kor, ketoisovalerate ferrodoxin-dependent reductase; Pfl, pyruvate-formate lyase; Pfor, pyruvate:ferredoxin oxidoreductase.

**FIGURE 4:**
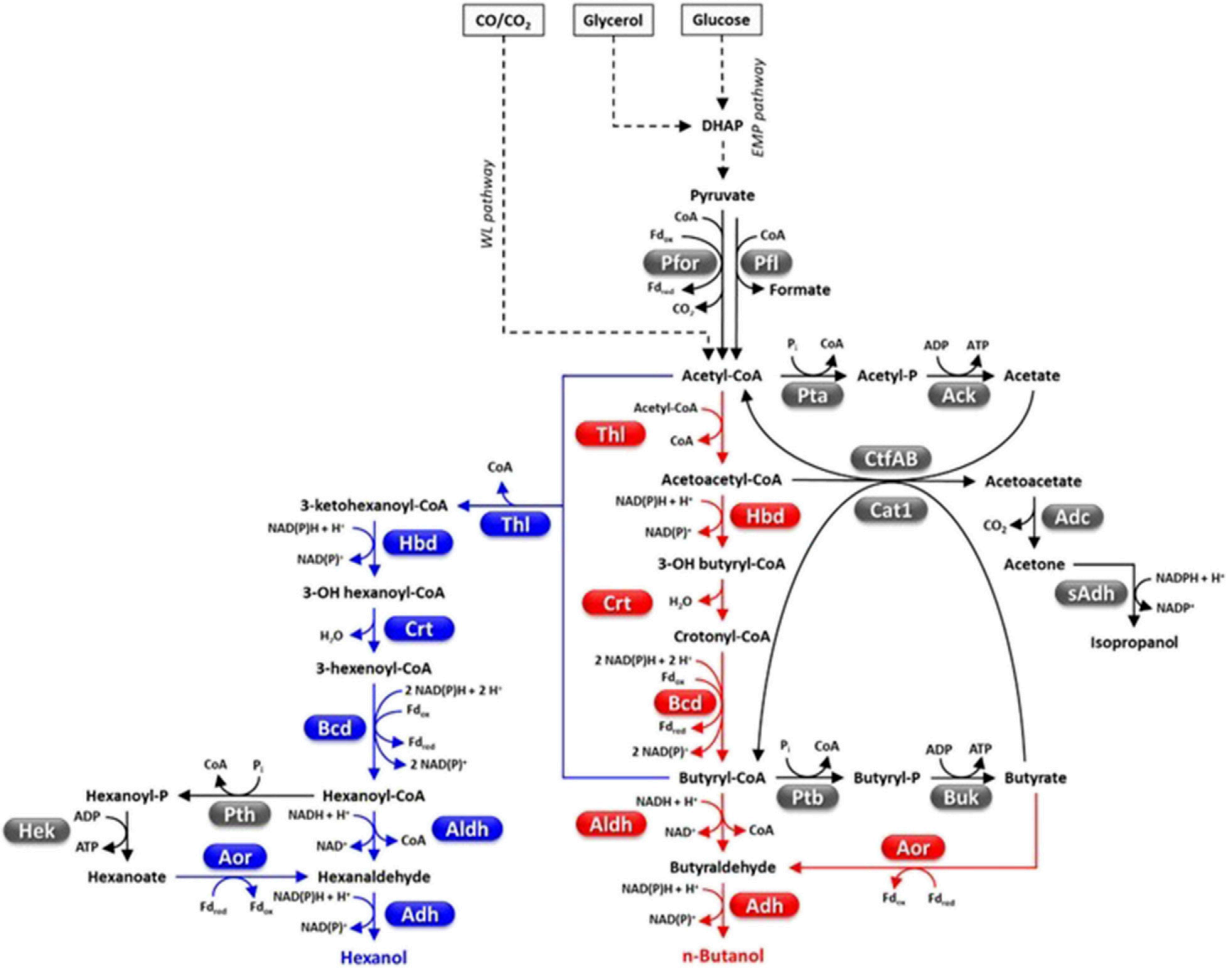
Fermentative pathways in Clostridia, production of C4-C6 alcohols. Abbreviations: Acetyl-P, acetyl phosphate; Ack, acetate kinase; Adc, acetoacetate decarboxylase; Adh, alcohol dehydrogenase; Aldh, aldehyde dehydrogenase; Aor, aldehyde ferredoxin oxidoreductase; Bcd, butyryl-CoA dehydrogenase; Buk, butyrate kinase; Butyryl-P, butyryl phosphate; Cat1, butyryl-CoA−acetate CoA transferase; Crt, crotonase; CtfAB, CoA transferase; DHAP, dyhydroxyacetone phosphate; Fd, ferredoxin; Hbd, 3-hydroxybutyryl-CoA dehydrogenase; Hek, hexanoate kinase; Pfl, pyruvate-formate lyase; Pfor, pyruvate ferredoxin oxidoreductase; Pta, phosphotransacetylase; Ptb, phosphotransbutyrylase; Pth, phosphotranshexanoylase; sAdh, secondary alcohol dehydrogenase; Thl, thiolase.

Clostridia include a large group of anaerobic gram-positive bacteria which have found large application in biomass biorefining (Cruz-Morales et al., 2019; Yang et al., 2022). Actually, the ABE (that stands for acetone, n-butanol and ethanol, in 3:6:1 ratio) fermentation of starch or sugar by *Clostridium acetobutylicum* was one of the largest fermentation industries until the 1960s when it was essentially replaced by cheaper oil-based technologies (Jones and Woods, 1986; Green, 2011; Jiang et al., 2015). Interest in biotechnological generation of n-butanol (hereinafter referred to as butanol) and other valuable chemicals has been revived in the last decades as a means to reduce dependence on fossil fuels, reduce CO_2_ emissions and ultimately improve the environmental sustainability of these productions (Azambuja and Goldbeck, 2020; Bao et al., 2020; Wen et al., 2020c; Ferreira et al., 2020; Nawab et al., 2020; Li et al., 2021). Within this framework, Clostridia are among the candidates with the greatest potential. A number of Clostridia can grow using inexpensive substrates such as lignocellulosic biomass (Mazzoli and Olson, 2020) or one carbon (C1) gases (CO, CO_2_) (Zhang et al., 2020b). *Clostridium* fermentation products include several compounds with important industrial application such as organic acids (e.g., lactate), short chain alcohols (e.g., ethanol, butanol, isobutanol, isopropanol), diols (e.g., 1,2-propanediol, 1,3-propanediol), acetone and H_2_ (Figures 1–4) (Altaras et al., 2001; Mazzoli, 2012; Wilkens et al., 2012; Mazzoli and Olson, 2020).

Development of universal systems for genetic manipulation of Clostridia (Minton et al., 2016; Yang et al., 2016; Wen et al., 2020d) has enabled significant enhancement of their natural potential as microbial cell factories (Charubin et al., 2018). This constantly expanding tool box also includes editing clostridial genome based on Clustered Regularly-Interspaced Short Palindromic Repeat (CRISPR)/cas (CRISPR associated) technology (Walker et al., 2020; Wilding-Steele et al., 2021; Husaini et al., 2023) or fine tuning gene expression by riboswitches (Marcano-Velazquez et al., 2019) or CRISPR interference (Ganguly et al., 2020). Consistently, significant improvement has been obtained as regards *Clostridium* production of a number of valuable chemicals and fuels such as ethanol (Tian et al., 2016; Hon et al., 2017), butanol (Re and Mazzoli, 2023), isobutanol (Higashide et al., 2011; Lin et al., 2015) and medium chain esters (Guo et al., 2023; Seo et al., 2023).

The very next sections will provide a survey of the main raw materials for *Clostridium* fermentation (Section 2) and the metabolic pathways involved (Section 3). The following sections will summarize research progress in production of some of the most valuable fuels and chemicals by Clostridia (Table 1), with special focus on strain improvement by metabolic engineering.

**TABLE 1:** Most recent estimation of economic parameters and applications of some top value chemicals produced by Clostridia.

No of C Atoms	Product	Price (US$/Kg)	Market size (US$ million)	Annual production (M Tons)	CAGR (evaluated period)	Application fields	References
—	Hydrogen	0.9–1.7	170,140	50	9.3% (2024–2030	Energy sector/electricity generation for industries and households, fuel sector	Wang and Yin (2021), Grand View Reserach (2023), Kindra et al. (2023), Worku et al. (2024)
C2	Ethanol	n.a.	89,100	≈110	4.8% (2020–2027)	Biofuels, food and beverages	Grand View Research (2024)
C3	1,2-PDO	1–2.2	373	1.36	1.6% (2020–2026)	Monomer for polymer synthesis (polyester resins)/antifreeze agent/liquid detergent, additive in cosmetics/food	Tao et al. (2021), Shrirame et al. (2023)
	1,3-PDO	3–3.5	424.5	0.1	9.1% (2023–2030)	Polymer synthesis (PTT), food preservative, miosturaizer in cosmetics/personal care, solvent	Cen et al. (2022), Agrawal et al. (2023), Nimbalkar and Dharne (2024)
	Isopropanol	n.a	2,650	2	8.2% (2023–2030)	Fuel industry, production of bulk chemicals (propylene), bio-plastics, solvent, antifreeze agents, disinfectants	Walther and François (2016), Grand View Research (2020)
	Lactic acid	1.3–4.0	1,300	≈0.3	12.4% (2023–2028)	Biodegradable polymers, food and beverages, personal care/cosmetics, pharmaceuticals	Klotz et al. (2017), MarketsandMarkets (2024)
C4	Butanol	0.4–2.9	8,400	n.a	6.2% (2023–2031)	Precursor of paints/polymers/plastics, biofuels	Transparency Market Research (2022), Re and Mazzoli (2023)
	Isobutanol	n.a	1,000	≈0.55	6.3% (2021–2030)	Biofuels, beverages, antiseptic, perfumes, paints, precursor of esters	Grand View Research (2022), Allied Market Research (2024)
	2,3-BDO	2–5	43,000	32	3.5% (2023–2031)	Additive to fuels, printing inks, fumigants, moistening agents and anti-freeze agents	Köpke et al. (2011), Koutinas et al. (2016), Mailaram et al. (2022), Transparency Market Research (2024)
C6	Hexanol	n.a	1,400	n.a	4.10% (2023–2030)	Fuel industry, solvent, plasticizer, pesticide, flavoring agent, chemical intermediate	Lauer et al. (2022a), Verified Market Research (2024)
C4-C8	Short/Medium Chain Esters	n.a	89,360	n.a	5.4% (2023–2033)	Fuels, solvents, flavors, food additives, fragrances	Future Market Insights (2024)

Abbreviations: 1,2-PDO, 1,2-propanediol; 1,3-PDO, 1,3-propanediol; 2,3-BDO, 2,3-butanediol; CAGR, compound annual growth rate; n.a., not available.

## 2 Feedstocks for *Clostridium* fermentation

Overall, Clostridia can ferment a wide range of substrates comprising soluble sugars (e.g., glucose, xylose, fructose, lactose, cellobiose), polysaccharides (e.g., starch, cellulose), glycerol and gaseous carbon compounds (CO, CO_2_) (Wang and Yin, 2021; Fernández-Blanco et al., 2023). Since feedstock expenditure may account for more than 70% of the total fermentation cost, the use of waste biomass is preferable to more expensive substrates such as pure sugars or edible crops, especially for producing bulk compounds such as fuels and platform chemicals (Gu et al., 2011; Abo et al., 2019; Rawoof et al., 2021). As a comparison, sugar costs about 460 US$/ton, pulp grade wood (a lignocellulosic biomass) can be calculated at 43–54 US$/ton of fermentable sugars and no cost can be referred to food waste (Nuss and Gardner, 2013; Gharehkhani et al., 2015; International Sugar Organization, 2019; Qureshi et al., 2020).

Carbohydrate-rich feedstocks include: i) lignocellulosic biomass; ii) (micro) algae biomass; iii) food waste; iv) municipal waste; v) agro-industrial effluents. Large availability and low cost of lignocellulosic feedstocks, such as wastes from agriculture (e.g., straws, stalks, wood fibers) and food processing (e.g., bagasse, mushroom compost) make it an ideal raw material for biorefining processes (Wang and Yin, 2021). However, this is offset by its complex composition (mainly consisting of cellulose, hemicellulose and lignin) and innate recalcitrance to biodegradation (Lynd, 2017). (Micro) algae biomass has gained considerable attention owing to its fast growth, no requirement of farmland, low demand for growth conditions and no or reduced lignin content which makes its hydrolysis and fermentation easier than plant biomass (Liu et al., 2012a; Ortigueira et al., 2015a; Ortigueira et al., 2015b; Fonseca et al., 2020). Globally, food waste (a very abundant biomass with high starch content) represents almost one-third of food produced for human consumption, and its disposal significantly contributes to greenhouse gas emission (Parthiba Karthikeyan et al., 2018; Qin et al., 2018; Zhang et al., 2020a; Su et al., 2022). Carbohydrate-rich raw materials also include some municipal wastes such as sewage sludge (Yin and Wang, 2016; Yin and Wang, 2019), paper waste (Liu et al., 2006) and garden wastes (leaves, branches) (Yang et al., 2019a). However, most of them have a complex composition (e.g., organics are mostly encapsulated in microbial cells in sewage sludge, paper and garden wastes are rich in lignocellulose) and generally need pretreatment prior to fermentation (Wang and Yin, 2018). Carbohydrate-rich agro-industrial effluents comprise sugarcane juice, molasses, cassava wastewater and cheese whey (Wang and Yin, 2021).

Glycerol is the main by-product (10% w/w) obtained by transesterification or saponification reactions aimed at producing oleochemicals such as biodiesel (Yang et al., 2012). The substantial increase in the biodiesel industry in the recent years has led to massive production of crude glycerol and drop in prices, hence, turned glycerol into a waste stream rather than a by-product (Ciriminna et al., 2014; Russmayer et al., 2019).

C1 gases (CO, CO_2_) are part of the greenhouse gases (mainly CO_2_) contributing to global warming and climate change (Canatoy et al., 2022). The use of these compounds as fermentation feedstocks can decrease their emission into the atmosphere by human activities. Among the industries that use fossil fuels for generating power and heat, steelmaking process emits about 50% of the carbon used as CO (Bengelsdorf et al., 2018). CO, CO_2_ (and H_2_) are also the major components of syn (thesis) gas which can be generated from natural gas, by gasification of coal, oil, biomass (e.g., agricultural and municipal waste) and by recycling used plastics (Köpke et al., 2010; Arslan et al., 2019; Zhang et al., 2020b). Syngas has extensively been used as a feedstock in the chemical industry, but this requires precise CO/H_2_ ratio and expensive gas purification from interfering contaminants (Köpke et al., 2010). Chemoautotrophic Clostridia are far more tolerant to such contaminants and already industrially used for ethanol production from these feedstocks (by companies such as Coskata, INEOS Bio, LanzaTech) (Köpke et al., 2010). However, low gas-liquid mass transfer rate (due to poor solubility of these gases in water) results in low cell densities and fermentation efficiency and is the main limit of this technology (Fernández-Blanco et al., 2023).

## 3 Heterotrophic and autotrophic fermentative pathways of Clostridia

Clostridia include bacteria with heterotrophic and autotrophic metabolism. In saccharolytic strains, glucose is generally converted to pyruvate through the Embden Meyerhof Parnas (EMP) pathway, since most Clostridia lack the oxidative part of the pentose phosphate pathway (Crown et al., 2011; Koendjbiharie et al., 2020; Foulquier et al., 2022). It is worth noting that clostridial EMP pathway may contain a number of atypical reactions with respect to the traditional glycolysis which affect electron distribution among the different redox cofactors [pyridine cofactors, ferredoxin (Fd)], cellular pools of energy carriers (e.g., adenine/guanine nucleotides, pyrophosphate) and pathway thermodynamics (Iddar et al., 2002; Zhou et al., 2013; Scott et al., 2019; Jacobson et al., 2020). Some heterotrophic Clostridia can also ferment more reduced substrates than carbohydrates such as glycerol which is converted to dihydroxyacetone phosphate (DHAP) and enters the EMP pathway (Yoo et al., 2016; Agu et al., 2019; Sarma et al., 2019).

Pyruvate can be fermented to a variety of compounds such as organic acids (e.g., acetate, butyrate, formate, lactate), short chain alcohols (e.g., ethanol, n-butanol, isobutanol), acetone, CO_2_ and H_2_ (Figures 1–4) (Mazzoli, 2012; Mazzoli and Olson, 2020). Metabolic flux distribution among fermentative pathways significantly differs from one strain to another and is affected by the growth conditions (e.g., the kind and amount of carbon sources, agitation, H_2_ partial pressure, pH, bioreactor operation mode) (Łukajtis et al., 2018; Yang et al., 2020; Wang and Yin, 2021; Fernández-Blanco et al., 2023; Julkipli et al., 2023). A major regulator of carbon flux distribution is the redox-responsive protein Rex (Wietzke and Bahl, 2012; Schwarz et al., 2017). Rex can affect gene transcription in response to changes of intracellular NADH/NAD^+^ ratio (Ravcheev et al., 2012) and is involved in modulating central carbon metabolism, solvent and organic acid production, H_2_ generation, tolerance to oxidative stress, biofilm formation, and sulfate and nitrate reduction (Hu et al., 2016; Sander et al., 2019). Furthermore, a network of enzymes known as ferredoxin:NAD oxidoreductases (Fnor) catalyze re-distribution of electrons deriving from substrate oxidation among redox cofactors (ferredoxin, NAD, NADP) and, finally, to the fermentation end-products (Mazzoli and Olson, 2020).

Some Clostridia can grow chemoautotrophically using CO and/or CO_2_ as the carbon source(s) (Liew et al., 2017; Zhang et al., 2020b) which are reduced to acetyl-CoA through the Wood–Ljungdahl (WL) pathway (Müller, 2003; Schuchmann and Müller, 2014). If CO_2_ is used as the sole carbon substrate, H_2_ is required as the reductant. The WL pathway requires eight reducing equivalents and one ATP. Energy is provided by specialized version of Fnor which is proton translocating reduced ferredoxin:NAD^+^ oxidoreductase (Rnf) and ATP synthase (which uses the proton gradient generated by Rnf for ATP synthesis). In most Clostridia growing autotrophically, acetyl-CoA is mainly converted to acetic acid but some strains such as *C. autoethanogenum*, *C. ragsdalei*, *C. ljungdahlii* and *C. carboxidivorans* can also produce other chemicals such as ethanol, butyrate, butanol, 2,3-butanediol (2,3-BDO), hexanoate, hexanol and lactate (Köpke et al., 2011; Arslan et al., 2019; Arslan et al., 2022) (Figures 1–4). In the latter strains, the production of solvents (e.g., ethanol) through gas fermentation generally occurs in two steps. First, CO/CO_2_ are converted to acids (usually acetic acid) in a step called acetogenesis, then the accumulated acids are reduced to alcohols (solventogenesis) (Arslan et al., 2019; Arslan et al., 2022). Biosynthesis of lactate and 2,3-BDO occurs through pyruvate formation (Figures 2, 3). In fact, in autotrophic Clostridia, pyruvate ferredoxin oxidoreductase (Pfor) can also catalyze acetyl-CoA reductive carboxylation to pyruvate (acetyl-CoA + CO_2_ + Fd_red_ → pyruvate + CoA + Fd_ox_) (Figures 2, 3), a reaction that is coupled to CO oxidation by CO dehydrogenase/acetyl-CoA synthase complex (Codh/Acs) (Furdui and Ragsdale, 2000).

## 4 Production of industrially relevant compounds by Clostridia

### 4.1 Hydrogen

Hydrogen gas (H_2_) is an optimal energy carrier featuring high energy content (122 kJ/g) and clean combustion product (Table 1) (Akhlaghi and Najafpour-Darzi, 2020). Biological production of H_2_ has gained attention over traditional technologies (e.g., steam reforming of CH_4_, coal gasification) (Valle et al., 2019; Akhlaghi and Najafpour-Darzi, 2020) because it does not rely upon usage of fossil fuels and has reduced CO_2_ emissions (Lepage et al., 2021). However, current technologies for biological production of H_2_ need to improve their yield and cost competitiveness (Lepage et al., 2021; Nirmala et al., 2023).

Bio-H_2_ production by the so called dark processes, that is anaerobic fermentation of organic compounds by a number of heterotrophic microbes (mainly bacteria), is generally considered more effective than light-driven processes (i.e., direct and indirect biophotolysis, photofermentation by means of photosynthetic microorganisms) (Das and Veziroglu, 2008; Mudhoo et al., 2011; Arizzi et al., 2021; Cao et al., 2022). Among microorganisms catalyzing dark fermentation, obligate anaerobes such as *Clostridium* spp. feature 2-fold higher maximum theoretical H_2_ yield (i.e., 4 mol/mol hexose) compared to facultative anaerobes (e.g., *Escherichia coli*, *Enterobacter* sp.) (Wang et al., 2011a). Clostridial genomes generally encode multiple hydrogenases (H_2_ases) likely involved in different functions (e.g., redox balancing, derivation of energy from H_2_ oxidation, proton respiration and/or proton-gradient build-up) (Calusinska et al., 2010; Arizzi et al., 2021). These include monomeric FeFe H_2_ases that catalyze proton reduction to H_2_ by oxidation of reduced ferredoxin or flavodoxin (Demuez et al., 2007; Flamholz et al., 2012; Therien et al., 2017; Morra, 2022) and multimeric electron-confurcating H_2_ases that catalyze reduction of protons to H_2_ via the oxidation of reduced ferredoxin and NAD(P)H (Figure 1) (Wang et al., 2013; Latifi et al., 2019). In Clostridia, reducing equivalents for proton reduction by H_2_ases mainly derive from glyceraldehyde-3-phosphate (GAP) oxidation by GAP dehydrogenase (Gapdh) and pyruvate oxidation by Pfor (Figure 1).

Metabolic engineering strategies aimed to increase H_2_ production in Clostridia have focused on different targets that include: i) overexpression of (native and/or heterologous) H_2_-producing enzymes and/or downregulation of uptake H_2_ases (Nakayama et al., 2008; Cha et al., 2016; Sarma et al., 2019; Son et al., 2021); ii) impairment of metabolic pathways competing for reducing equivalents (e.g., production of ethanol, butyrate, formate, lactate) (Wang et al., 2011a; Jiang et al., 2011; Lo et al., 2015; Rydzak et al., 2015); iii) improvement of substrate catabolism (Sarma et al., 2019; Son et al., 2021; Kim et al., 2023); iv) optimization of electron/redox metabolism (Lo et al., 2017; Nguyen et al., 2018; Foulquier et al., 2022). It is worth remembering that maximum H_2_ yield can be obtained when sugars are fermented to acetate (glucose + 4 ADP + 4 P_i_ → 2 acetate + 2 CO_2_ + 4 ATP + 2 H_2_O + 4 H_2_), while it is lower when more reduced products (e.g., propionate, butyrate, lactate, ethanol) are accumulated (Wang et al., 2011a; Wang et al., 2021b; Ortigueira et al., 2015a; Islam et al., 2015). Studies aiming at improving monosaccharide catabolism include diversion of glucose towards the pentose phosphate (PP) pathway (Son et al., 2021). In fact, glucose fermentation through the PP pathway could increase the maximum theoretical H_2_ yield by 33% (1 glucose + 3.33 ADP + 3.33 P_i_ → 1.67 acetate + 2.67 CO_2_ + 3.33 ATP + 5.33 H_2_) (Singh et al., 2019).

So far, most of these investigations achieved limited H_2_ yield enhancement (generally comprised between 15% and 80%) with H_2_ yield still lower than 2 mol/mol hexose in most engineered strains (Mazzoli et al., 2024). One likely reason is that only one gene has generally been down- or upregulated in each engineered strain. For instance, in Clostridia multiple hydrogenase-encoding genes are present, with different roles and expression levels, and the balance of their activity can be a key to improved performances (Land et al., 2020; Arizzi et al., 2021; Morra, 2022; Fasano et al., 2024). Combination of multiple advantageous metabolic modifications (e.g., overexpression of evolving H_2_ases, elimination of alternative pathways, optimization of sugar metabolism) in one strain seems among the most obvious implementation. More extensive application of adaptive laboratory evolution could be an additional tool that avoid or implement complex rational genetic engineering to tackle all these issues and enhance H_2_ production of microorganisms (Tunca et al., 2023). Along with strain optimization, the use of improved fermentation conditions (e.g., pH, temperature, substrate concentration, H_2_ partial pressure), mode (e.g., continuous fermentation) and bioreactor configuration is pivotal (Cai et al., 2013; Wang et al., 2014; Łukajtis et al., 2018; Son et al., 2021; Julkipli et al., 2023). From this standpoint, the improvement of systems for reducing H_2_ partial pressure in the bioreactor (e.g., stirring the growth medium, sparging the growth medium with inert gas, removing gas by a vacuum pump, selectively removing H_2_ by active membranes) is essential for overcoming thermodynamic barriers of biological H_2_ production. The Δ_r_G’^m^ (i.e., the change in Gibbs free energy associated with a metabolic reaction/pathway when all the reactants have a concentration of 1 mM) for the production of H_2_ by oxidation of glucose to acetate (through the EMP pathway) can vary from −205.1 to 0.2 kJ/mol for dissolved H_2_ concentration ranging from 10^–9^ M–1 M (Flamholz et al., 2012). Promising results have also been reported through the development of processes based on syntrophic microbial chains, such as two-step fermentation (e.g., dark fermentation + photofermentation) (Ramprakash et al., 2022) or co-cultures (e.g., H_2_ producing bacteria + bacteria able to perform anaerobic respiration) (Zhang et al., 2023b) providing complete oxidation of *Clostridium* fermentation by-products. Improvement of H_2_ yield obtained by combining dark- and photo-fermentation was significantly higher (≈100–200%) (Ramprakash et al., 2022) than those reported for co-cultures (≈30–45%) (Zhang et al., 2023b). The efficiency of syntrophic co-cultures could be enhanced within the framework of microbial electrolysis cells in which additional electric voltage is used to increase H_2_ yield from organic compound oxidation (Bora et al., 2022).

In summary, the high potential of Clostridia for H_2_ production has been so far improved to a limited extent by a number of studies employing metabolic engineering. More intense efforts in this direction are desirable. Process optimization (e.g., reduction of H_2_ partial pressure, synthrophic microbial chains for complete substrate oxidation) is also necessary for achieving suitable efficiency for industrial application.

### 4.2 Ethanol

Ethanol is the most broadly produced biofuel today, with over 16 billion gallons produced in the United States alone in 2019 (Table 1) (Ethanolrfa, 2019). It is typically blended with gasoline for use in spark-ignited engines (10% in the United States, 27% in Brazil), but can also be catalytically upgraded to longer-chain fuel molecules, such as jet fuel and gasoline (Hannon et al., 2020; Mazzoli and Olson, 2020). 3.1 EJ/year of ethanol is currently obtained from sugar cane (30%) and cereals (50%) (Lynd et al., 2017). However, projected future global demand of bioethanol and the need for more massive reduction of greenhouse gas emission will require a significant contribution by bioethanol derived from other feedstocks (e.g., lignocellulose, CO_2_) (Wang et al., 2015; Lynd et al., 2017). Today, bioethanol is primarily produced by fermentation of mono- or disaccharides using *Saccharomyces cerevisiae* or *Zymomonas mobilis*. Nonetheless, these organisms cannot directly grow on cheap feedstocks such as lignocellulose or syngas (Himmel et al., 2007). Substantial research has been dedicated to develop recombinant cellulolytic yeast or *Z. mobilis* strains, yet the maximum cellulosic ethanol titer obtained through direct biomass fermentation by these strains (≤10 g/L) is far lower than what is generally considered as necessary for commercial application (titer = 40 g/L, yield = 1.8 mol/mol hexose) (Dien et al., 2003; Kojima et al., 2013; Todhanakasem et al., 2019; Anandharaj et al., 2020). *Clostridium thermocellum* and *Thermoanaerobacterium saccharolyticum* have been important alternative microbial paradigms for one-step production of ethanol from cellulose or hemicellulose, respectively (Figure 1) (Mazzoli and Olson, 2020). On the other hand, acetogenic Clostridia such as *C. ljungdahlii* (Phillips et al., 1993) and *C. ragsdalei* (Sun et al., 2018) have been investigated as promising ethanol producers through C1-gas fermentation.

*C. thermocellum* naturally produces low ethanol yield (typically 12%–34% of the theoretical maximum, i.e., 2 mol/mol hexose) (Olson et al., 2015), but extensive metabolic engineering efforts have significantly increased this efficiency (Mazzoli and Olson, 2020). These studies have included: i) elimination of pathways that compete for carbon and electron flux (namely, production of acetate, lactate, formate, and H_2_) (Biswas et al., 2015; Papanek et al., 2015; Rydzak et al., 2015; Holwerda et al., 2020); ii) improvement of electron metabolism (Hon et al., 2017; Hon et al., 2018; Lo et al., 2017); iii) improvement of glycolytic flux (Deng et al., 2013; Zhou et al., 2013; Tian et al., 2017b; Hon et al., 2022); iv) overexpression of autologous and heterologous genes for ethanol production (e.g., alcohol dehydrogenase, aldehyde dehydrogenase, pyruvate decarboxylase) (Tian et al., 2017a; Zheng et al., 2017; Hon et al., 2018). Eventually, these efforts led to strains which can produce ethanol at titers of 25–30 g/L and high yields (75%–80% of the theoretical maximum) (Olson et al., 2023). Still, this is too low for commercial application. In particular, ethanol titer in these strains seems to be limited by ethanol tolerance. The latter has been improved in the wild type *C. thermocellum* to 50–80 g/L (Williams et al., 2007; Brown et al., 2011; Shao et al., 2011). However, a recent investigation has highlighted a tradeoff between ethanol tolerance and production, namely, *C. thermocellum* strains with enhanced ethanol tolerance generally show decreased ethanol production (Olson et al., 2023). The main reason for *C. thermocellum* inhibition by ethanol accumulation seems to be related to redox, i.e., NADH/NAD^+^ ratio, imbalance (which affect alcohol dehydrogenase and GAP dehydrogenase activities) but other yet elusive mechanisms could be involved.

*T. saccharolyticum* is a thermophilic anaerobic bacterium which can ferment xylan (the main polymer in hemicellulose) and all the most abundant monosaccharides of plant biomass (e.g., glucose, mannose, xylose, galactose, and arabinose) although it cannot use cellulose (Herring et al., 2016). The wild-type *T. saccharolyticum* accumulates ethanol as its main fermentation product (yield = 1 mol/mol xylose) but also significant amounts of acetate and lactate (Shaw et al., 2008). Disruption of genes involved in production of acetate (phosphotransacetylase, *pta*, and acetate kinase, *ack*) and adaptation to high xylose concentration resulted in homoethanologenic phenotype with a maximum ethanol titer = 37 g/L (Shaw et al., 2008). In a following study, higher performing *T. saccharolyticum* strains were obtained by introducing additional genetic modifications such as the expression of a urease and the disruption of an operon involved in exopolysaccharide biosynthesis (Herring et al., 2016). These strains were able to produce up to 70 g/L of ethanol from a mixture of cellobiose and maltodextrin. However, much lower ethanol titer (26 g/L) was obtained by fermentation of a hemicellulose extract (Herring et al., 2016). Interestingly, co-culture of *C. thermocellum* and *T. saccharolyticum* (or other hemicellulolytic microbes such as (*Thermoanaerobacterium thermosaccharolyticum* and *Herbinix* spp.) has been performed which enabled one-pot fermentation of both the cellulose and hemicellulose components of plant biomass (He et al., 2011; Jiang et al., 2013; Froese et al., 2019; Beri et al., 2020).

As mentioned above, acetogenic Clostridia have been studied for ethanol production via C1-gas fermentation. This generally occurs in two steps, that is acetogenesis precedes ethanol formation (solventogenesis) (Fernández-Blanco et al., 2023). Gas fermenting Clostridia can produce ethanol through two pathways that is: i) reduction of acetyl-CoA by aldehyde/alcohol dehydrogenase or; ii) reduction of acetate to acetaldehyde by acetaldehyde ferredoxin oxidoreductase (Aor) and then reduction of acetaldehyde by alcohol dehydrogenase (Figure 1) (Zhang et al., 2020b). Ethanol production is triggered by stress conditions limiting cell growth such as acidic pH and/or lack of nutrients (Daniell et al., 2012; Fernández-Naveira et al., 2016; Al-Shorgani et al., 2018). The highest ethanol titer reported through C1-gas fermentation (48 g/L) was achieved in 1993 by Philips and co-workers by using a *C. ljungdahlii* strain growing on syngas (Phillips et al., 1993). This result was obtained after 560 h fermentation in a stirred tank bioreactor with cell recirculation, using an optimized growth medium and high gas-liquid mass transfer. This study indicated that a medium with pH range of 4.0–4.5 promoted ethanol accumulation. However, far lower ethanol titers were obtained in more recent investigations (Fernández-Blanco et al., 2023). As far as we know, the highest ethanol titer (16.25 g/L) reported by the latest studies was obtained through syngas fermentation by *C. ragsdalei* in a medium supplemented with poultry litter biochar (Sun et al., 2018). As regards strain improvement by metabolic engineering, significant increase of ethanol production (50%–180%) by *C. autoethanogenum* or *C. carboxidivorans* was obtained by either inactivation (Liew et al., 2017) or overexpression (Lu et al., 2019) of *adhE* genes (encoding bifunctional alcohol/aldehyde dehydrogenase). However, the maximum ethanol titers obtained through autotrophic growth of these strains were ≤ 3 g/L (Liew et al., 2017; Cheng et al., 2019).

In conclusion, Clostridia provide promising paradigms for production of ethanol from low-cost feedstocks, such as lignocellulose and C1-gas. Research progress is at a more advanced stage as regards lignocellulose fermentation (also in reason of the higher number of studies), while investigation of C1-gas fermentation still needs substantial efforts.

### 4.3 C3 compounds

#### 4.3.1 1,2-propanediol

1,2-propanediol (1,2-PDO) is a bulk chemical with applications in antifreeze agents, cosmetics, nutrition, medicine and polyester resins (Table 1) (Siebert and Wendisch, 2015). Currently, 1,2-PDO is mostly produced through chemical hydration of fossil-derived propylene which leads to a racemic mixture of R- and S-1,2-PDO (Tao et al., 2021). Fermentative production of 1,2-PDO benefits from the use of renewable feedstocks and can generate pure 1,2-PDO stereoisomers (Tao et al., 2021). However, native bacterial producers of 1,2-PDO (*Prevotella*, *Salmonella*, *Klebsiella, Corynebacterium*, *Clostridium*) show low yield and productivity, hindering their application in industrial processes (Turner and Robertson, 1979; Badia et al., 1985; Cameron and Cooney, 1986; Siebert and Wendisch, 2015).

Natural 1,2-PDO producing Clostridia include *Clostridium* sp. AK1 (Ingvadottir et al., 2018), *C. beijerinckii* DSM 6423 (Diallo et al., 2019), *C. phytofermentans* (Petit et al., 2013), *C. sphenoides* and *C. thermosaccharolyticum* (Tran-Din and Gottschalk, 1985; Cameron and Cooney, 1986; Altaras et al., 2001). Clostridia can produce 1,2-PDO through two alternative metabolic pathways, the deoxyhexose (DXH) or the methylglyoxal (MGL) pathway (Figure 2) (Tao et al., 2021). The main limit of the DHX pathway is that it requires expensive feedstocks such as L-fucose and L-rhamnose (Figure 2) (Tran-Din and Gottschalk, 1985; Cameron and Cooney, 1986). The MGL route allows the conversion of a much larger panel of cheaper sugars (e.g., glucose, fructose, mannose, galactose, xylose, arabinose, lactose or cellobiose), however, suffers from possible accumulation of toxic intermediates (e.g., methylglyoxal) which limit bacterial growth and 1,2-PDO production (Tao et al., 2021). Efforts have been made to find cheaper natural sources of L-rhamnose, such as the macroalgae *Ulva lactuca* which contains L-rhamnose and D-glucose as the major sugars (Diallo et al., 2019). *C. beijerinckii* DSM 6423 was able to accumulate up to 5.96 g/L 1,2-PDO by fermenting (DHX pathway) a *U. lactuca* hydrolysate with a yield of 0.41 g/g of rhamnose (Diallo et al., 2019). *C. thermosaccharolyticum* HG-8 is equipped with the MGL pathway and is currently the highest performing 1,2-PDO producer among Clostridia (Sánchez-Riera et al., 1987). This strain was reported to produce 1,2-PDO from a variety of feedstocks (e.g., glucose, xylose, mannose, cellobiose or whey permeate) up to a titer = 9.05 g/L (yield = 0.20 g/g hexose) (Table 2) (Sánchez-Riera et al., 1987). As far as we know, no study has attempted to increase 1,2-PDO production in Clostridia by metabolic engineering strategies which, to date, have mainly targeted *E. coli* (Tao et al., 2021).

**TABLE 2:** Comparison between production of top value chemicals by Clostridia (bold) and other high performing microorganisms.

No of C Atoms	Product	Microorganism(s)	Fermentation mode	Feedstock	Titer (g/L)	Yield (g/g)	Productivity (g/L/h)	Reference
—	Hydrogen	*Enterobacter cloacae* DM11	Batch + reduced H_2_ partial pressure	Glucose	n.a	0.044	3.4 *10^–5^	Mandal et al. (2006)
		***Clostridium perfringens* ATCC 13124**	**Batch + pH regulation**	**Glucose**	**n.a**	**0.044**	**n.a**	Wang et al. (2014)
C2	Ethanol	*Zymomonas mobilis/Saccharomyces cerevisiae*	Various	Glucose	up to 100	0.4–0.5	up to 80	Panesar et al. (2006)
		**Engineered *Clostridium thermocellum***	**Continuous fermentation**	**Cellulose**	**29.9**	**0.29**	**n.a**	Holwerda et al. (2020)
		**Engineered *Thermoanaerobacterium saccharolyticum***	**Batch**	**Cellobiose + maltodextrine**	**70.0**	**0.46**	**n.a**	Herring et al. (2016)
C3	1,2-PDO	Engineered *Escherichia coli*	Fed-batch	Glucose	17.3	0.18	0.72	Niu et al. (2019)
		Engineered *Escherichia coli*	Batch	Glucose	5.6	0.21	0.078	Clomburg and Gonzalez (2011)
		***Clostridium thermosaccharolyticum* HG-8**	**Batch**	**Glucose**	**9.05**	**0.20**	**n.a**	Cameron and Cooney (1986)
	1,3-PDO	Engineered *Escherichia coli*	Fed-batch	Glucose	135	0.51	3.5	Nakamura and Whited (2003)
		***Clostridium butyricum***	**Fed-batch**	**Glycerol**	**93.7**	**0.52**	**3.3**	Wilkens et al. (2012)
	Isopropanol	Engineered *Escherichia coli*	Fed-batch	Glucose	40.1	0.73	0.66	Inokuma et al. (2010)
		Engineered *Escherichia coli*	Batch	Glucose	8.0	0.45	0.12	Shen and Liao (2013)
		**Engineered *Clostridium acetobutylicum***	**Batch**	**Glucose**	**8.8**	**0.14**	**0.30**	Collas et al. (2012)
	Lactic acid	Lactic acid bacteria/*Bacillus* sp	Batch	Glucose	up to 190	≈0.9	2.5–4.4	Alves de Oliveira et al. (2018), Rawoof et al. (2021)
		**Evolved *Caldicellulosyruptor* sp**	**Batch**	**Microcrystalline cellulose**	**70**	**0.85**	**1.0**	Svetlitchnyi et al. (2022)
C4	Butanol	*Engineered Escherichia coli*	Fed-batch + gas stripping	Glucose	30	0.29	0.18	Shen et al. (2011)
		**Engineered *Clostridium beijerinckii***	**Fed-batch + gas stripping**	**Glucose**	**157.7**	**0.31**	**0.76**	Ezeji et al. (2004)
		**Engineered *Clostridium acetobutylicum***	**Continuous fermentation + extractive distillation**	**Glucose**	**550**	**0.35**	**0.14**	Nguyen et al. (2018)
	Isobutanol	Engineered *Escherichia coli*	Batch	Glucose	22.0	0.35	n.a	Atsumi et al. (2008)
		**Engineered *Clostridium thermocellum***	**Batch**	**Cellulose**	**5.4**	**0.17**	**0.072**	Lin et al. (2015)
	2,3-BDO	*Klebsiella oxytoca*	Fed-batch	Glucose	150	n.a	4.21	Ma et al. (2009)
		***Clostridium ljungdahlii***	**Fed-batch**	**CO**	**16.9**	**n.a**	**0.061**	Zhu et al. (2020)
C6	Hexanol	Engineered *Escherichia coli*	Batch	Glucose	0.47	n.a	n.a	Machado et al. (2012)
		***Clostridium carboxidivorans***	**Batch**	**CO**	**1.90**	**n.a**	**n.a**	Oh et al. (2022)
		***Clostridium carboxidivorans***	**Fed-batch +** ***in situ*** **product extraction**	**CO + ethanol**	**8.45**	**n.a**	**n.a**	Oh et al. (2023)
C8	Butyl butyrate	*Engineered Escherichia coli*	Batch + *in situ* product extraction	Glucose + butyrate	≈0.005	n.a	2*10^–4^	Layton and Trinh (2016)
		**Engineered *Clostridium tyrobutyricum***	**Batch**	**Cassava starch**	**26.8**	**n.a**	**0.19**	Guo et al. (2024)
		**Engineered *Clostridium tyrobutyricum***	**Fed-batch +** ***in situ*** **product extraction**	**Mannitol**	**63**	**0.17**	**0.31**	Guo et al. (2023)

n.a., not available.

#### 4.3.2 1,3-propanediol

1,3-propanediol (1,3-PDO) has various industrial uses in cosmetics (e.g., solvent, moisturizer), food (e.g., preservative) and polymer synthesis (e.g., polytrimethylene terephthalate) (Table 1). Since 2003, 1,3-PDO has mainly been produced via fermentation of corn-derived glucose by an engineered *E. coli* strain through a collaboration of DuPont, Genencor and Tate & Lyle group (Agrawal et al., 2023). Advantageously, a number of microorganisms including some Clostridia can naturally produce 1,3-PDO through glycerol fermentation (Figure 2) (Nimbalkar and Dharne, 2024). When glycerol is the only carbon source, a part of it is dehydrated and reduced to 1,3-PDO, however, another part is oxidized to DHAP which enters the glycolytic flux, thus providing the reducing power for generating 1,3-PDO (Figure 2). Glycerol oxidative metabolism leads to accumulation of by-products such as organic acids (mainly acetic, butyric, and lactic), alcohols (e.g., ethanol, butanol, 1,2-propanediol, 2,3-butanediol), H_2_ and CO_2_, depending on the species and culture conditions (Nimbalkar and Dharne, 2024). The most studied natural producers of 1,3-PDO are *Klebsiella pneumoniae* and *Clostridium butyricum* owing to their high glycerol consumption rate and 1,3-PDO production (titer up to 100 g/L, yield ≈ 0.5 g/g, productivity ≈ 2 g/L/h) (Wilkens et al., 2012; Zhu et al., 2022). Natural 1,3-PDO producing-Clostridia offer many advantages with respect to *K. pneumoniae* since they are non-pathogenic, produce lower amounts of by-products and require cheaper fermentation systems (Yazdani and Gonzalez, 2007). For instance, *C. butyricum* and other *Clostridium* strains biosynthesize a vitamin B12-independent glycerol dehydratase (GDHt, glycerol → 3-hydroxypropionaldehyde + H_2_O) (Figure 2), hence, do not require exogenous supplementation of this expensive vitamin in the growth medium (Raynaud et al., 2003). Yet, optimization of 1,3-PDO production by *C. butyricum* is hindered by low tolerance to product accumulation, large amounts of by-products (e.g., butyric acid, acetic acid) and lack of efficient gene modification tools (Yang et al., 2019b).

This has prompted research on other native or engineered 1,3-PDO producing Clostridia such as *C. acetobutylicum* (González-Pajuelo et al., 2005), *C. beijerinckii* (Wischral et al., 2016; Schoch et al., 2023), *C. diolis* (Otte et al., 2009; Li et al., 2020a) or *C. perfringens* (Guo et al., 2017) for which maximum 1,3-PDO titers range between 40-84 g/L. Particularly noteworthy are the results obtained with an engineered *C. acetobutylicum* (González-Pajuelo et al., 2005). In fact, the *C. acetobutylicum* ATCC824 DG1 mutant (unable to produce solvents and sporulate) overexpressing the genes encoding *C. butyricum* GDHt and 1,3-propanediol oxidoreductase (Pdor, 3-hydroxypropionaldehyde + NADH + H^+^ → 1,3-PDO + NAD^+^) (Figure 2) was able to ferment glycerol and produce 1,3-PDO as the major product in fed-batch fermentation (titer = 84 g/L 1,3-PDO, yield = 0.53 g/g, maximum productivity = 1.70 g/L/h). Interestingly, a two-step fermentation involving *C. acetobutylicum* was developed which was able to convert glucose or molasses to 1,3-PDO (Mendes et al., 2011). In the first fermentation, the recombinant *S. cerevisiae* HC42 (adapted to grow on high glucose concentration) converted sugars into glycerol, which was subsequently fermented to 1,3-PDO by a 1,3-PDO hyperproducing *C. acetobutylicum* DG1 (final titer = 25.5 g/L, yield = 0.56 g/g of glycerol and 0.24 g/g of glucose, maximum productivity = 0.16 g/L/h). This study is paradigmatic of alternative strategies for extending the panel of feedstocks for fermentative production of 1,3-PDO by Clostridia beyond glycerol.

#### 4.3.3 Lactic acid

Lactic acid (LA) is among the chemicals with the largest worldwide industrial demand by sectors that include the food, cosmetic, pharmaceutical industry and the synthesis of biodegradable solvents and plastics (e.g., polylactide) (Table 1) (Abdel-Rahman et al., 2011; Abdel-Rahman et al., 2013; Alves de Oliveira et al., 2018). About 90% of global LA production is obtained by microbial fermentation producing pure L- or D-LA enantiomer (chemical synthesis generates a racemic mixture) (Abdel-Rahman et al., 2013; Alves de Oliveira et al., 2018) which is required for plastic polymer synthesis (Abdel-Rahman and Sonomoto, 2016), and food and pharmaceutical applications (Jem et al., 2010). Currently, industrial production of LA mainly rely on lactic acid bacteria (LAB) (Sauer et al., 2008) based on their high LA yield, productivity and GRAS (generally regarded as safe) status (Abdel-Rahman et al., 2013; Rawoof et al., 2021). However, LAB also have drawbacks such as limited acid tolerance, requirement for complex nutrients (amino acids, nucleotides, vitamins) and inability to directly ferment cheap feedstocks (e.g., lignocellulose). This has stimulated research on alternative microbial platforms for LA production (Poudel et al., 2016; Tarraran and Mazzoli, 2018). Within this perspective, a major advantage of using Clostridia for LA production is the ability of (hemi) cellulolytic strains to directly ferment lignocellulosic biomass. One-step fermentation of lignocellulose to LA could dramatically decrease the cost of LA production and its current dependence from food crops (Nuss and Gardner, 2013; Gharehkhani et al., 2015; International Sugar Organization, 2019; Qureshi et al., 2020).

So far, the number of studies aimed at enhancing LA production in natural (hemi) cellulolytic microorganisms is limited. Research on bacteria such as *C. thermocellum* (Lo et al., 2015; Mazzoli et al., 2020), *C. bescii* (Williams-Rhaesa et al., 2018), *Thermoanaerobacter mathranii* (Yao and Mikkelsen, 2010), *T. saccharolyticum* (Zhou et al., 2015), *Thermoanaerobacterium aotearoense* (Yang et al., 2013) and *Thermoanaerobacterium thermosaccharolyticum* (Bhandiwad et al., 2013) has suggested valuable metabolic engineering strategies to increase LA accumulation in these strains. These include impairment of alternative fermentative pathways (e.g., production of H_2_, acetate, ethanol, formate) (Mazzoli, 2020), increasing the expression of lactate dehydrogenase (Ldh) (Figure 2) (Williams-Rhaesa et al., 2018; Mazzoli et al., 2020), or engineering the redox state of the cell (Ravcheev et al., 2012; Lo et al., 2017; Sander et al., 2019). Studies on *C. thermocellum* (Mazzoli et al., 2020), *Caldicellulosiruptor saccharolyticus* (Willquist and van Niel, 2010) and *Thermoanaerobacter ethanolicus* (Bryant, 1991) have indicated that the catalytic activity of their Ldh is modulated by a number of compounds such fructose-1,6-bisphosphate, nicotinamide cofactors (e.g., NADH, NAD^+^) and/or, energy carriers (e.g., ATP, PP_i_), which need to be taken into account in metabolic engineering strategies.

One of the main hurdles in engineering LA hyper-production in anaerobic (hemi) cellulolytic microbes is their limited acid tolerance (Yang et al., 2013; Mazzoli et al., 2022; Svetlitchnyi et al., 2022). Growth at regulated pH through base addition was essential for major increase of the LA production of a *Thermoanaerobacterium aotearoense* strain deficient in acetate production (Yang et al., 2013). This growth condition resulted in nearly homolactic fermentation (LA yield = 0.93 g/g glucose) with a final LA titer up to 47 g/L (Yang et al., 2013). As base addition is complicated and expensive at the industrial scale, use of strains with high acid tolerance is recommended (Singhvi et al., 2018). Ideally, a microbial host should tolerate pH conditions around the pK_a_(s) of the produced acid (typically in the range 3–5 for organic acids) (Skoog et al., 2018). So far, *C. thermocellum* and *Caldicellulosyruptor* sp. strains have been obtained by adaptive laboratory evolution which have increased tolerance to LA (Mazzoli et al., 2022; Svetlitchnyi et al., 2022). An evolved *Caldicellulosyruptor* sp. strain showed more than 10-fold increase in LA titer and was able to produce up to 70 g/L LA through batch fermentation of microcrystalline cellulose (Svetlitchnyi et al., 2022). This titer is comparable to that obtained through fermentation of cellulosic biomass hydrolysate by lactic acid bacteria (Rawoof et al., 2021). However, it should be noted that pH regulation of *Caldicellulosyruptor* sp. cultures was still necessary (Svetlitchnyi et al., 2022). In fact, acid stress depends on both the decrease of pH and the nature of the acid, i.e., more hydrophobic carboxylic acids generally are more toxic (Jarboe et al., 2013; Wilbanks and Trinh, 2017). Irrespective of the strategy used (random mutagenesis, strain evolution, rational engineering), no research could lower the acidic pH limit allowing a microorganism to grow beyond 0.5 pH unit such as in the case of the anaerobic cellulolytic bacteria *Clostridium cellulovorans* and *Fibrobacter succinogenes* (Wu et al., 2017; Wen et al., 2020a; Mazzoli, 2021). More intense effort in this direction is needed as regards LA hyper-producing cellulolytic Clostridia.

#### 4.3.4 Overview of C3-compound production by Clostridia

Among the C3 compounds considered here, production of 1,3-PDO by *C. butyricum* is the most established (Table 2), although the lack of efficient genetic tools hampers strain improvement (e.g., increase product tolerance, reduce by-products formation) and increase in process efficiency which has stimulated research on other clostridial strains (e.g., *C. acetobutylicum*). A few engineered clostridial strains have shown promising potential for direct fermentation of lignocellulose to lactic acid although their limited acid tolerance constitutes a major issue. As reported also for other microorganisms (Table 2), 1,2-PDO production by Clostridia is intrinsically hindered by the high cost of substrates or by accumulation of toxic intermediates which need to be addressed (e.g., by metabolic engineering).

### 4.4 C4 compounds

#### 4.4.1 Butanol and isobutanol

##### 4.4.1.1 Improving butanol production in natural solventogenic Clostridia

Butanol has high potential as a drop-in fuel (namely, it can be fed to spark ignited engines without any modification) which adds to its applications as paint, polymer and plastic precursor (Table 1) (Gu et al., 2011; Campos-Fernández et al., 2012; Jiang et al., 2015). ABE fermentation is still the most economically viable route for biobutanol production, yet, it is affected by several drawbacks especially under industrial conditions: i) high cost of feedstock and substrate inhibition; ii) low butanol titer (≤20 g/L), yield (≈0.33 g/g), and productivity (<0.5 g/L/h); iii) important formation of by-products which increase butanol purification costs; iv) poor understanding of *Clostridium* physiology (Green, 2011; Gu et al., 2011; Abo et al., 2019; Li et al., 2020c). Current research aimed to improve butanol production by native solventogenic Clostridia includes: i) enhancing butanol yield, titer and tolerance; ii) expanding fermentation substrates to low-cost feedstocks (e.g., food waste, lignocellulosic biomass and C1-gases) (Zhang et al., 2020a; Fernández-Blanco et al., 2023; Re and Mazzoli, 2023).

Four *Clostridium* species, *C*. *acetobutylicum*, *C*. *beijerinckii*, *C*. *saccaroperbutylacetonicum* and *C*. *saccharoacetobutylicum*, can biosynthesize significant butanol amounts (Figure 4) (Keis et al., 2001; Huang et al., 2010). The growth of these strains develops through three phases that is acidogenesis, solventogenesis and sporogenesis which is subjected to complex metabolic regulation whose molecular details are still poorly understood (Li et al., 2020c). The exponential phase is characterized by accumulation of acids (mainly acetic and butyric acid), ATP and reduced pyridine cofactors. Growth medium acidification and accumulation of ATP, NAD(P)H and possibly other metabolites (e.g., butyryl-phosphate, formic acid) contribute to shift the metabolism towards solvent (acetone, butanol, ethanol) production and is accompanied by acid re-assimilation (Zhao et al., 2005; Wang et al., 2011b; Li et al., 2020c). Biomass production, non-assimilation of acids, and accumulation of other carbohydrates contribute to lower the actual butanol yield with respect to the theoretical maximum (1 mol/mol of glucose, that is 0.41 g/g) (Li et al., 2020c).

Significant enhancement of butanol production by natural solventogenic *Clostridia* has been obtained by: i) strain improvement (by mutagenesis, metabolic engineering, adaptive laboratory evolution); ii) optimization of the growth medium; iii) optimization of the fermentation process (e.g., high cell density fermentation, use of *in situ* product recovery techniques) [extensively reviewed by Li et al. (2020c)]. Further improvement of bacterial strains through mutagenesis is hampered by the complex and unknown metabolic/phenotypic changes generated by random gene mutations (Palsson and Zengler, 2010). Rational metabolic engineering has targeted genes involved in: i) butanol biosynthetic pathway; ii) pathways competing for carbon and electrons; iii) butanol tolerance (illustrated in Section 4.4.1.4); iv) redox homeostasis; v) energy homeostasis; vi) regulation of acidogenesis-solventogenesis shift and solvent production (Wietzke and Bahl, 2012; Nguyen et al., 2018; Li et al., 2020c; Dai et al., 2021; Re and Mazzoli, 2023).

The acetyl-CoA-butanol production pathway is intrinsically hampered by its high NADH consumption (5 mol/mol butanol) (Figure 4) and is not very exergonic (∆_r_G’^m^ = −50.1 ± 13.7 KJ/mol) (Flamholz et al., 2012). Metabolic engineering strategies have been used to enhance the cellular NADH levels by up-regulating NADH formation pathways [e.g., by overexpressing heterologous ferredoxin-NAD(P) oxidoreductase] (Qi et al., 2018) or reducing NADH consumption by the butanol pathway (Lee et al., 2012; Nguyen et al., 2018; Qi et al., 2018; Li et al., 2019; Li et al., 2020b). As regards the latter strategy, increased butanol flux in *C. acetobutylicum* was obtained by replacing NADH-dependent enzymes (e.g., 3-hydroxybutyryl-CoA dehydrogenase, Hbd; alcohol-aldehyde dehydrogenase, AdhE) with NADPH-dependent counterparts (Figure 4) (Lee et al., 2012; Nguyen et al., 2018). *C. acetobutylicum* thiolase (CaThl, 2 acetyl-CoA → acetoacetyl-CoA + CoA, ∆_r_G’^m^ = 25.0 ± 1.7 kJ/mol) is among the most critical nodes of the butanol pathway. CaThl is subject to redox-switch, namely, oxidized cell conditions lead to enzyme inactivation and repression of butanol (and butyric acid) biosynthesis (Flamholz et al., 2012; Kim et al., 2015). Furthermore, CaThl is inhibited by low concentration of CoA (Nguyen et al., 2018). Engineering CaThl protein to alleviate feedback inhibition (Mann and Lütke-Eversloh, 2013) or avoid enzyme inactivation by oxidized conditions (Kim et al., 2015), or replacing CaThl with *E. coli* thiolase (AtoB) (that shows higher catalytic efficiency, lower sensitivity to CoA and is not redox-switch modulated) (Nguyen et al., 2018) increased Thl activity and/or butanol flux to varying degrees (18%–64%). Recently, early activation (that is during acidogenesis) of butanol production leading to increased titer has accidentally been obtained by trying to engineer a complete WL pathway in *C. acetobutylicum* (Jang et al., 2023).

The impairment of biosynthetic pathways for alternative fermentation products (e.g., acetate, acetone, butyrate, ethanol, lactate) (Figures 1–4) has generally decreased by-product accumulation and enhanced butanol yield (Lee et al., 2012; Nguyen et al., 2018) but sometimes generated undesired phenotypes (e.g., defective acid assimilation or growth rate) (Li et al., 2020c). Elimination of acetone production (a non-fuel solvent with high corrosivity) (dos Santos Vieira et al., 2019) has been reported to increase acetate (and ethanol) generation and decrease butanol accumulation (Jiang et al., 2009; Lehmann et al., 2012). Instead, overexpression of secondary-alcohol dehydrogenases (sAdh) (coupled with upregulation of acetone pathway and disruption of butyrate production) in *C. acetobutylicum* resulted in efficient conversion of acetone to isopropanol (up to 8 g/L) (Dusséaux et al., 2013; dos Santos Vieira et al., 2019; Zhang et al., 2023a). This strategy generated isopropanol, butanol, ethanol (IBE) mixture that, differently from the traditional ABE fermentation, can be directly applied to spark-ignition engines without the need to remove by-products (Peralta-Yahya and Keasling, 2010; Zhang et al., 2023a). Engineering of *C. acetobytylicum* aldehyde/alcohol dehydrogenases has been used to increase substrate specificity for butyryl-CoA and increase butanol over ethanol production (Cho et al., 2019).

So far, the largest increases in butanol titer and/or productivity have been obtained by engineering the fermentation process, that is by using fed-batch or continuous configuration, and/or using immobilized cells and/or using *in situ* product recovery (e.g., pervaporation, adsorption, liquid–liquid extraction, gas stripping, vacuum fermentation) (Nguyen et al., 2018; Li et al., 2020c), leading to alleviation of substrate and/or product inhibition. So far, the highest butanol titer (550 g/L) was obtained by an engineered *C. acetobutylicum* strain in a continuous high cell density bioreactor with *in situ* alcohol extractive distillation (Table 2) (Nguyen et al., 2018). However, these improved fermentation systems are generally associated with higher technical complexity and cost (Nguyen et al., 2018; Li et al., 2020c).

Few natural Clostridia can produce low butanol titer (≤3.6 g/L) through direct fermentation of cellulose or hemicellulose (Mendez et al., 1991; Virunanon et al., 2008; Li et al., 2018). The development of a recombinant cellulolytic *C. acetobutylicum* has been hampered by severe issues in heterologous cellulase expression (Mingardon et al., 2011; Kovács et al., 2013; Willson et al., 2016). However, the replacement of the inactive catalytic module of *C. acetobutylicum* cellulase Cel48A with a homologous domain from *C. cellulolyticum* Cel48F enabled direct fermentation of phosphoric acid swollen cellulose (although no butanol titer, yield or productivity was reported) (Soucaille et al., 2010). (Hemi)cellulolytic and solvent-producing Clostridia have been used to develop artificial consortia or generate fusant strains (Re and Mazzoli, 2023) leading to production of 11–14 g/L butanol through direct fermentation of lignocellulosic feedstocks (Wen et al., 2014; Begum and Dahman, 2015; Jiang et al., 2020). These titers are close to those generated through fermentation of lignocellulose hydrolysates by solventogenic Clostridia (Gu et al., 2011) but still significantly lower than those obtained by conversion of starch or soluble sugars (≈20 g/L) (Gu et al., 2011; Wen et al., 2014; Abo et al., 2019). Among C1-gas fermenting Clostridia, only *C. carboxidivorans* can naturally produce low amounts (≤2 g/L) of butanol in addition to hexanol and ethanol (Fernández-Naveira et al., 2017; Fernández-Blanco et al., 2023). Recently, metabolic engineering has resulted in minor increase of butanol titer (18%) in this bacterium (Cheng et al., 2019). Higher butanol titer (2.6–6.8 g/L) was obtained by mixotrophic growth (i.e., using both sugars and C1-gas as growth substrates), or by natural or artificial microbial co-cultures (Fernández-Blanco et al., 2023).

##### 4.4.1.2 Engineering butanol production in non-native *Clostridium* hosts

Engineering butanol production in non-native hosts has at least two possible advantages: i) no production of other solvents which allows simplified downstream processing for product purification; ii) selection of hosts that can naturally ferment low-cost substrates (e.g., lignocellulose, CO_2_). Much interest has been attracted by the hyper-butyrate producer *C. tyrobutyricum* (Bao et al., 2020). In fact, this microorganism has high metabolic flux toward butyryl-CoA and a high butanol tolerance (>15 g/L) (Yu et al., 2011). In addition, *C. tyrobutycum* has rarely been reported to be subjected to bacteriophage infection, which is a common issue of industrial ABE fermentation (Bao et al., 2020). A butanol hyper-producing *C. tyrobutyricum* was developed by overexpressing the *C. acetobutylicum* bifunctional acetaldehyde-alcohol dehydrogenase AdhE2 and disrupting the gene encoding butyrate:acetate CoA transferase (Cat1, which catalyzes butyrate production) which can generate 26.2 g/L butanol through glucose fermentation (Figure 4) (Lee et al., 2016; Zhang et al., 2018). This butanol titer is actually higher than those reported for native butanol producers. Hence, *C. tyrobutyricum* appears to be a microbial platform with high potential for butanol production, at a level similar or higher than other non-native butanol producers such as *E*. *coli* (Shen et al., 2011) or the yeast *Arxula adeninivorans* (Kunze and Haehnel, 2011).

Butanol production has mainly been engineered in three cellulolytic Clostridia, *C. cellulolyticum* (Gaida et al., 2016) *C. cellulovorans* (Yang et al., 2015) and *C. thermocellum* (Tian et al., 2019b). Reduced or imbalanced biosynthesis of butanol pathway enzymes, enzyme instability, insufficient availability of co-factors and unfavorable reaction thermodynamics have probably contributed to a variable extent to modest butanol titer (<0.5 g/L) in *C. cellulolyticum* and *C. thermocellum* (Gaida et al., 2016; Tian et al., 2019b). Fewer genetic modifications enabled butanol production in *C. cellulovorans* (it is naturally equipped with a butyryl-CoA biosynthetic pathway) (Yang et al., 2015; Wen et al., 2019) which likely contributed to its higher butanol titer (4.96 g/L) from lignocellulosic biomass (Wen et al., 2020b). Similar metabolic engineering strategies used for ABE fermenting strains [e.g., impairing alternative fermentative pathways, enhancing NAD(P)H availability, dysregulating redox homeostasis] are likely to succeed also in further improving butanol formation in *C. cellulovorans* (and other cellulolytic Clostridia) (Bao et al., 2021; Re and Mazzoli, 2023). However, the efficiency of genetic tools for manipulating *C. cellulovorans* is still limited (Wen et al., 2017). Apart from the native ability to produce butanol of *C. carboxidivorans*, the butanol pathway has been engineered in another C1-gas fermenting *Clostridium,* namely, *C. ljungdahlii* (Köpke et al., 2010; Lauer et al., 2022b). However, butanol titer < 0.2 g/L was obtained by this engineered strain through autotrophic growth. An alternative approach to bioconvert C1-gases into alcohols with longer chain than C2 consists in co-culturing gas fermenting strains with chain-elongating strains (Fernández-Blanco et al., 2023). Different gas fermenting Clostridia (e.g., *C. aceticum*, *C. autoethanogenum*, *C. carboxidivorans*, *C. ljungdahlii*) have been co-cultured with the chain-elongating model strain *Clostridium kluyvery* resulting in production of C4-C8 alcohol mixtures (maximum alcohol titer generally ≤ 1 g/L). (Diender et al., 2016; Richter et al., 2016; Bäumler et al., 2022; Fernández-Blanco et al., 2022). In fact, *C. kluyvery* is able to elongate the chain of acetate and ethanol (produced by acetogenic Clostridia) to butyrate, hexanoate and octanoate by using reverse β-oxidation. On the other side, acetogenic strains can reduce these fatty acids to their corresponding alcohols (Figure 4). One of the main limitations of this approach is that acetogenic bacteria usually have an optimum pH for growth close to 6, while that of *C. kluyveri* is close to neutrality (Fernández-Blanco et al., 2023).

##### 4.4.1.3 Production of isobutanol

Isobutanol is an attractive vehicle fuel with energy density similar to butanol and higher octane number, which is advantageous for blending into gasoline (Chen and Liao, 2016). Moreover, isobutanol can be dehydrated to isobutene, which can then be converted to C8-C12 alkenes to be used as jet fuel (Lin et al., 2015). Additional uses of isobutanol and its derivatives are as solvents, additives in paints, ink ingredients, and extractants for organic compounds (Table 1) (Nawab et al., 2024). A few microorganisms such as *Lactococcus lactis*, *S. cerevisiae*, *Pichia pastoris* and *Candida* sp. can naturally produce very little isobutanol amounts (≤0.44 g/L) (Nawab et al., 2024). In *S. cerevisiae* and lactic acid bacteria, isobutanol is biosynthesized through the diversion of 2-ketoisovalerate (an intermediate of valine and isoleucine biosynthesis) which is decarboxylated to isobutyraldehyde by 2-ketoisovalerate decarboxylase and finally reduced (Figure 3) (Hazelwood et al., 2008). Higher levels of isobutanol have been produced by engineered microbial platforms (Nawab et al., 2024) with studies targeting *Corynebacterium glutamicum* (Yamamoto et al., 2013; Hasegawa et al., 2020) and *E. coli* (Atsumi et al., 2008) reporting the highest titers (≥20 g/L) through glucose fermentation. However, much lower isobutanol titer (1.88 g/L) was obtained through fermentation of cheaper feedstocks such as pretreated corn stover (Minty et al., 2013).

Advantageously, native production of higher isobutanol levels (1.6 g/L) has been observed in the cellulolytic bacterium *C. thermocellum* (Holwerda et al., 2014). Isobutanol biosynthetic pathway in *C. thermocellum* differs from that found in lactic acid bacteria or *S. cerevisiae* in that 2-ketoisovalerate is converted to isobutyraldehyde in two steps (Figure 3) (Lin et al., 2015). First, ferredoxin-dependent ketoisovalerate reductase (KOR) catalyzes oxidative decarboxylation of 2-ketoisovalerate to isobutyryl-CoA by which is then reduced to isobutyraldehyde (Figure 3). Isobutanol production of *C. thermocellum* was enhanced by overexpressing autologous acetohydroxy acid synthase (Als), keto acid reductoisomerase (Kari) and dihydroxy acid dehydratase (Dhad) and introducing *L. lactis* 2-ketoisovalerate decarboxylase (KivD) (Figure 3) (Lin et al., 2015). In optimized growth conditions, the most efficient engineered *C. thermocellum* strain produced 5.4 g/L of isobutanol from cellulose, which corresponds to 41% of the theoretical yield (Lin et al., 2015). Since this concentration is close to the isobutanol tolerance of the wild type *C. thermocellum* (Tian et al., 2019a), it is likely that isobutanol production in this microorganism is limited by tolerance. Less successful engineering of isobutanol production (titer ≤ 0.66 g/L) was reported in other cellulolytic Clostridia, i.e., *C. cellulolyticum* (Higashide et al., 2011) and *C. cellulovorans* (Wen et al., 2022). Recently, an original approach was reported which consisted in producing a mixture of butanol and isobutanol through direct fermentation of alkali extracted deshelled corn cobs (Wen et al., 2022). To this aim, artificial consortia were developed that included engineered strains of *C. cellulovorans* and *C. beijerinckii* which were able to generate up to 1.05 g/L isobutanol and 6.22 g/L butanol in the same fermentation (Wen et al., 2022). Efforts to engineer the isobutanol pathway into autotrophic Clostridia have also been reported (Weitz et al., 2021). However, fermentation of a syngas mixture (50% CO, 45% H_2_, 5% CO_2_) by an engineered *C. ljungdahlii* strain could only produce ≈ 70 mg/L isobutanol (Weitz et al., 2021).

##### 4.4.1.4 Improving butanol/isobutanol tolerance

Butanol (and isobutanol) toxicity is among the main issues of biological production of this chemical(s). These compounds are inherently more noxious than other established biofuels, such as ethanol, owing to their higher hydrophobicity (Heipieper et al., 2007; Wilbanks and Trinh, 2017). The toxicity of butanol is mainly due to impairment of structure and functions of biological membranes, dissipation of proton motive force and ATP pools and protein denaturation (Bowles and Ellefson, 1985; Tomas et al., 2004; Alsaker et al., 2010; Venkataramanan et al., 2015). Even native butanol-producing strains, e.g., *C. acetobutylicum*, can typically tolerate up to 1%–2% v/v butanol (Huang et al., 2010; Nicolaou et al., 2010).

Increasing microbial tolerance to butanol/isobutanol is a key aspect for enhancing their biological production especially as regards final titer. To this aim, responses to butanol stress have been studied in several microbial species that showed the implication of a very complex network of mechanisms only partially understood (Re and Mazzoli, 2023). Hence, increasing butanol tolerance by targeted gene manipulation (e.g., overexpression of protein chaperones) has so far attained only limited results. Random approaches (e.g., random mutagenesis, genome shuffling, adaptive evolutionary engineering) proved to be more suitable strategies to select for multiple-gene trait combinations conferring higher butanol resistance such as for mutant *C. acetobutylicum* strains able to tolerate up to 3%–4% (v/v) butanol (Liu et al., 2012b; Liu et al., 2013). Interestingly, the adaptive evolution strategy used to develop a butanol-hypertolerant *C. thermocellum* also enhanced tolerance to isobutanol to the same extent (i.e., 15 g/L), suggesting that similar physiological mechanisms allow cells to cope with both compounds (Tian et al., 2019a). Interestingly, most of the strains characterized by increased tolerance (and equipped with butanol pathway) also showed higher butanol production which highlights the importance of this line of research in improving biobutanol production (Re and Mazzoli, 2023).

Metabolic engineering strategies targeting global gene regulators involved in stress response could advantageously contribute to future development of butanol/isobutanol hypertolerant Clostridia (Jones et al., 2016; Mazzoli, 2021; Xu et al., 2021). Recent studies have indicated that small non-coding RNAs (sRNAs) and RNA chaperones (e.g., Hfq) have important roles in the ability of microorganisms to tolerate a variety of stresses such as butanol exposure (Venkataramanan et al., 2013; Jones et al., 2016; Sun et al., 2017; Costa et al., 2021).

#### 4.4.2 2,3-butanediol

2,3-butanediol (2,3-BDO) plays a critical role in numerous industrial sectors (Table 1) (Köpke et al., 2011). 2,3-BDO is an important starting material in the production of solvents like methyl-ethyl-ketone (MEK) and 1,3-butadiene, which are essential for creating fuel additives, resins, rubbers, printing inks, and lubricating oils. Additionally, 2,3-BDO is used in cosmetics and food industry and as antifreezing agent (Soltys et al., 2001). Nowadays, 2,3-BDO is mostly produced through cheap petrochemical processes, although a pilot plant for its biological production using *Klebsiella oxytoca* and *Paenibacillus polymyxa* was operating during the World War II (Blackwood et al., 1949; Celińska and Grajek, 2009). The most efficient natural producers of bio-2,3-BDO include *Paenibacillus polymyxa*, *Klebsiella pneumoniae, K*. *oxytoca, P*. *polymyxa, Serratia marcescens*, *Enterobacter aerogenes* and *S*. *cerevisiae* (Köpke et al., 2011). In particular, *Klebsiella* species show fast growth, can use a wide variety of simple sugars and produce up to 150 g/L 2,3-BDO through fed-batch fermentation of glucose (Köpke et al., 2011; Hakizimana et al., 2020). 2,3-BDO biosynthesis is primed by condensation of two pyruvate units to form acetolactate which is subsequently decarboxylated to acetoin (by α-acetolactate decarboxylase, Ald) and reduced to 2,3-BDO by acetoin reductase (Acr) (Figure 3) (Xiao and Xu, 2007; Caspi et al., 2008).

Although *C. acetobutylicum* is naturally equipped with acetoin biosynthetic pathway, overexpression of *C. beijerinckii* Acr led to modest 2,3-BDO production (≈2 g/L) (Siemerink et al., 2011). Instead, some C1-gas fermenting Clostridia such as *C*. *autoethanogenum*, *C. ljungdahlii*, *C. ragsdalei* can naturally produce 2,3-BDO (Köpke et al., 2011; Ricci et al., 2021). Nonetheless, autotrophic growth on CO-rich steel mill waste gas (44% CO, 32% N_2_, 22% CO_2_, and 2% H_2_) led to accumulation of small amounts of 2,3-BDO (<0.2 g/L) especially as compared to the predominant metabolic end products (1.7–1.9 g/L acetate, 0.9–1 g/L ethanol) (Köpke et al., 2011). A recent *in silico* study has suggested some metabolic engineering strategies for increasing 2,3-BDO biosynthesis by *C. autoethanogenum* (Ghadermazi et al., 2022). However, so far, the most significant progress in 2,3-BDO production by gas-fermenting Clostridia has been obtained through optimization of the growth medium, substrate composition and fermentation conditions (Zhu et al., 2020; Ricci et al., 2021). Improved gas-liquid mass transfer, gas mixture composition (CO:CO_2_ 4:1) and growth medium (Zinc and Iron supplementation) was able to significantly shift carbon flux of *C. ljungdahlii* from acetate/ethanol to 2,3-BDO production up to a titer ≈ 2 g/L (batch fermentation) (Ricci et al., 2021). Use of fed-batch fermentation technology with pH and gas (CO) pressure control dramatically increased 2,3-BDO titers up to ≈ 17 g/L (Zhu et al., 2020). It is worth noting that pH regulation is essential for energy conservation in *C. ljungdahlii* since it affects the Rnf-ATPase responsible for ATP generation (Zhu et al., 2020). It is likely that further optimization of the bioreactor configuration (e.g., continuous fermentation, optimized gas flow rate, cell recycling) can enable further enhancement of 2,3-BDO production by gas fermentation (Simpson et al., 2009; Simpson et al., 2014; Ricci et al., 2021).

#### 4.4.3 Overview of C4-compound production by Clostridia

Extensive research has been dedicated to improving butanol production by ABE fermenting strains and non-native butanol producers. Although native solventogenic Clostridia are still the most established microorganisms for industrial production of butanol, other engineered strains are promising alternative candidates on traditional substrates (e.g., *C. tyrobutyricum*) or lignocellulosic feedstocks (e.g., *C. cellulovorans*). More limited progress has been reported as regards the generation of isobutanol through direct fermentation of lignocellulose or C1-gases. Yet, a major challenge in the biological production of butanol/isobutanol is the development of hyper-tolerant strains which still requires substantial efforts. Despite the high commercial interest in 2,3-BDO, the levels so far produced by Clostridia are modest both from gaseous substrates and sugars.

### 4.5 Hexanol

Hexanol has different industrial applications (e.g., solvent, pesticide, flavoring agent, platform chemical) (Table 1). Currently, it is mainly obtained by petrochemical production systems, although it can also be generated through fermentation of sugars or gaseous substrates by a number of microorganisms, such as *C. carboxydivorans* (Lauer et al., 2022b). *C. carboxydivorans* can condense acetyl-CoA units to generate hexanoyl-CoA which is then reduced to hexanol (Figure 4) (Shen et al., 2017; Lauer et al., 2022b). *C. carboxidivorans* can produce hexanol also through the reduction of hexanoate by Aor (Wirth and Dürre, 2021). The highest hexanol titers obtained so far through CO fermentation by the wild-type *C. carboxidivorans* are 1.4–1.9 g/L (Shen et al., 2017; Oh et al., 2022). Other Clostridia such as *C. kluyvery* instead convert hexanoyl-CoA mainly into hexanoic acid (caproic acid) and produce only traces of hexanol (Figure 4) (Shen et al., 2017; Lauer et al., 2022b). However, as mentioned in the Section 4.4.1.2., *C. kluyvery* has been used as chain-elongating partner in co-cultures with gas-fermenting Clostridia (e.g., *C. carboxidivorans*, *C. ljungdahlii*) for fermenting C1 substrates into butanol-hexanol-octanol mixtures (Richter et al., 2016; Fernández-Blanco et al., 2023).

Recently, hexanol production was engineered in another gas fermenting *Clostridium* (*C. ljungdhalii*) (Lauer et al., 2022b). Two sets of genes encoding the acetyl-CoA-to-hexanoyl-CoA pathway of *C. kluyvery,* and the gene coding for the aldehyde-alcohol dehydrogenase AdhE2 of *C. acetobutylicum* (for the reduction of hexanoyl-CoA to hexanol) were integrated into the *C. ljungdahlii* chromosome (Figure 4). The engineered *C. ljungdahlii* showed improvement of both butanol and hexanol production (Lauer et al., 2022b). Fermentation in 2 L bioreactor, with continuous CO_2_-H_2_ supplementation and pH regulation (pH = 6) resulted in the production of 0.122 g/L hexanol. Limited hexanol titer was attributed to inefficient biosynthesis of some enzymes of the acetyl-CoA-to-hexanoyl-CoA pathway. Therefore, additional genes from *C. carboxidivorans* (encoding thiolase, crotonase, 3-hydroxybutyryl-CoA dehydrogenase and butyryl-CoA dehydrogenase complex) (Figure 4) were introduced in the genome of the engineered *C. ljungdahlii*. The final strain produced 0.251 g/L hexanol through fermentation of 20% CO_2_, 80% H_2_ gas mixture (Oh et al., 2022).

As for other products obtained through gas fermentation, significant research towards higher hexanol production has been focused on optimizing the fermentation process such as growth conditions, media composition, gas composition or supply (Oh et al., 2022). Substantial improvement was obtained by using *in situ* extraction of hexanol, which reduces hexanol accumulation in the fermentation medium (Kottenhahn et al., 2021; Oh et al., 2023). It is worth remembering that hexanol is even more toxic than butanol because of its longer carbon chain (Heipieper et al., 2007). Supplementation of 1 g/L hexanol is enough for reducing *C. ljungdahlii* growth and 5 g/L caused total growth cessation (Oh et al., 2023). Supplementation of small quantities of a biocompatible extractant (i.e., oleyl alcohol) and ethanol (as a precursor) to *C. carboxidivorans* P7 cultures growing on CO enabled production of 8.45 g/L hexanol, which is the highest titer reported so far (Oh et al., 2023).

### 4.6 Medium (C4-C8) chain esters

Short- and medium-chain (C2-C12) esters such as ethyl acetate, butyl acetate, isobutyl acetate and butyl butyrate, have a broad range of application as flavors, fragrances, pharmaceuticals, green solvents and advanced biofuels (Table 1) (Lee and Trinh, 2020; Wang et al., 2021a). The traditional methods for synthesizing short- and medium-chain fatty acid esters are mainly based on concentrated sulfuric acid-mediated esterification of acids and alcohols and are affected by serious health and environmental issues (Cull et al., 2000; Jermy and Pandurangan, 2005). Alternative chemical strategies based on ionic liquid catalysis can reduce these problems to some extent, but they are expensive and not stable (Tankov et al., 2017). In nature, microbes and plants can biosynthesize several esters which encounters the increasing consumer preference for natural and sustainable products (Lee and Trinh, 2020; Seo et al., 2021). Some yeasts and lactic acid bacteria can naturally form esters but with limited efficiency (Abeijón Mukdsi et al., 2009; Kruis et al., 2018). This has stimulated research aimed to engineer more efficient producers (Rodriguez et al., 2014; Kruis et al., 2017).

Clostridia can synthesize a variety of volatile organic acids (e.g., acetic and butyric acid) and alcohols (e.g., ethanol, butanol and isobutanol) (Tracy et al., 2012; Cho et al., 2015; Lin et al., 2015). Therefore, significant amounts of acyl-CoA, organic acids, and alcohols are available in cells that can serve as precursors for ester generation. Biological synthesis of esters is mainly catalyzed by esterases/lipases (alcohol + acid → ester + H_2_O) or by alcohol acyltransferases (AAT, alcohol + acyl-CoA→ester + CoA) (Kruis et al., 2019; Noh et al., 2019). In a number of studies, exogenous lipases have been supplemented to clostridial fermentation broth to convert acids and alcohols to esters (Cui et al., 2020; Wang et al., 2021a). The main issue of these approaches is the cost of exogenously added lipases (Wang et al., 2021a). Alternatively, overexpression of heterologous lipases in Clostridia has proven to be challenging (Wen et al., 2020c). The AAT-dependent pathway is more thermodynamically favorable (Flamholz et al., 2012; Noh et al., 2019). Overexpression of heterologous AATs has enabled different mesophilic Clostridia (e.g., *C. acetobutylicum*, *C. diolis*, *C. beijerinckii, C. saccharoperbutylacetonicum*) to produce esters such as butyl acetate and butyl butyrate from glucose or xylose (Horton and Bennett, 2006; Noh et al., 2018; Li et al., 2020a; Fang et al., 2020; Feng et al., 2021). Efforts have been made also for producing esters from cheaper feedstocks. By introducing an engineered heterologous chloramphenicol acetyltransferase and inactivating endogenous esterases, *C. thermocellum* was able to produce C4-C8 esters (e.g., ethyl acetate, ethyl isobutyrate, isobutyl acetate, and isobutyl isobutyrate) through direct fermentation of cellulose (Seo et al., 2023). As far as we know, only one study has reported engineering medium chain ester production in a gas fermenting *Clostridium* (namely, *C. autoethagenum*) resulting in accumulation of traces of ethyl-acetate and butyl-acetate (Dykstra et al., 2022).

One of the key aspects of engineering ester production in Clostridia relies on overproduction of stable AATs with desired substrate specificity (Seo et al., 2021). Most characterized AATs derive from plants or yeasts which results in poor biosynthesis, solubility and/or thermostability in prokaryotes (Horton and Bennett, 2006; Noh et al., 2018; Li et al., 2020a; Fang et al., 2020). Studies have been dedicated to optimize AATs structure and/or biosynthesis (Seo et al., 2021). In addition, limited knowledge is available on substrate specificities of AATs (Noh et al., 2018; Kruis et al., 2019). Both these aspects were tackled to develop an ester overproducing *C. saccharoperbutylaceticum* (Feng et al., 2021). In addition, systems metabolic engineering was used to increase availability of NADH and ester precursor(s) (namely, acetyl-CoA). The most efficient strain was able to produce up to 20.3 g/L butyl acetate (and 0.9 g/L butyl butyrate) through glucose fermentation and up to 17.8 g/L butyl acetate by fermentation of corn stover hydrolysate (Feng et al., 2021). Few metabolic modifications were able to convert *C. tyrobutyricum* (an efficient butyrate producer) into high performing butyl butyrate producing strain, namely, enhancing butyryl-CoA pathway (by upregulating CtfAB encoding CoA transferase, Figure 4), engineering butyryl-CoA reduction to butanol (by expressing *C. acetobutylicum* AdhE2), and introducing the wild strawberry alcohol acyltransferase (VAAT) (Guo et al., 2023). Additional reducing power for ester production was supplied by using a reduced growth substrate, that is mannitol, instead of glucose. Fed batch fermentation of mannitol at regulated pH and supplementation of hexadecane for *in situ* extraction of esters led to ≈ 63 g/L butyl butyrate (yield = 0.21 mol/mol, theoretical maximum is 0.5 mol/mol) (Table 2) (Guo et al., 2023). This is by far the highest medium chain ester titer obtained by microbial fermentation. Additional introduction of heterologous α-amylase and α-glucosidase enabled the engineered *C. tyrobutyricum* to produce high amounts of butyl butyrate (26.8 g/L) through batch fermentation of starch from a non-food crop (i.e., cassava) (Guo et al., 2024). As mentioned above for other hydrophobic products (e.g., butanol, hexanol), *in situ* extraction of esters significantly contributed to increase fermentation efficiencies of the engineered strains by limiting toxicity linked to product accumulation in the growth medium (Fang et al., 2020; Feng et al., 2021; Guo et al., 2023; Guo et al., 2024).

In conclusion, the variety of acyl-CoA, acids and alcohols produced by Clostridia confers them the ability to produce a panel of commercially interesting medium-chain esters in high amounts. Yet, challenges of this research area reside in efficient expression of AATs with desired substrate specificity and improving the availability of carbon precursors and reducing power.

## 5 Conclusion

The studies summarized in the present review illustrate the key role of Clostridia in the development of sustainable biomass biorefining processes. Clostridia are already the reference cell factories for fuels such as butanol and among the most promising microbial platforms for production of valuable chemicals (e.g., ethanol, lactate, H_2_, hexanol, medium chain esters) through direct fermentation of low cost biomasses (e.g., lignocellulose, C1-gases) (Table 2). Significant progress has also been reported as regards clostridial production of 1,2-PDO and 1,3-PDO, whose titers are not far from those obtained by the highest performing microorganisms (e.g., engineered *E. coli*) (Table 2). However, for the full deployment of *Clostridium* potential in commercial applications further advances at multiple levels are necessary.

Extensive investigations on the physiology and metabolism of Clostridia over the last decades have revealed peculiar characteristics (e.g., atypical pathway reactions, sophisticated regulation of enzyme activity) and subtle differences between the different strains which affect carbon and electron flux and pathway thermodynamics. We believe that further advances in detailed understanding the metabolic network of Clostridia will be major sources of inspiration for improving current metabolic engineering strategies. To this aim, further development of genetic tools for manipulating Clostridia is also necessary, especially for strains which are recalcitrant to the currently available methods (e.g., *Clostridium diolis*) or which shows low genetic tractability (e.g., *C. cellulovorans*).

Apart from strain improvement by metabolic engineering (enhancement of product yield, titer, productivity; increase of tolerance to toxic products), research aimed to optimize the fermentation process (e.g., growth media and conditions, fermentation mode, bioreactor configuration, use of syntrophic co-cultures) including extraction and purification of products will be essential to achieve the efficiency required by industrial application. Coping with the different feedstocks (e.g., insoluble solids such as lignocellulose versus gaseous substrates) and products (e.g., acids versus hydrophobic solvents versus gaseous products) described in the present review will certainly require far different process optimization. The efficiency of gas fermentation in inherently limited by the low solubility of CO/CO_2_ in water. Developing suitable systems for improving gas-liquid mass transfer is among the priority of this research area. Accumulation of acids (e.g., lactic acid) or solvents (e.g., butanol, hexanol, medium chain esters) reduces *Clostridium* fermentation efficiency due to limited tolerance of cells these chemicals. Both strain improvement by metabolic engineering and process engineering (e.g., pH regulation, *in situ* recovery of solvents) should synergistically provide a cost sustainable solution. As regards biological production of H_2_, the improvement of systems for reducing H_2_ partial pressure in the bioreactor (e.g., stirring the growth medium, sparging the growth medium with inert gas, removing gas by a vacuum pump, selectively removing H_2_ by active membranes) is essential for overcoming thermodynamic barriers of biological H_2_ production. We are confident that thanks to the contribution of these different approaches a rapid improvement of *Clostridium*-mediated biorefinery processes suitable for their industrial application will be possible.
